# Host plant affects morphometric variation of *Diaphorina citri* (Hemiptera: Liviidae)

**DOI:** 10.7717/peerj.2663

**Published:** 2016-11-03

**Authors:** Thomson M. Paris, Sandra A. Allan, David G. Hall, Matthew G. Hentz, Gabriella Hetesy, Philip A. Stansly

**Affiliations:** 1 Agriculture Research Service, United States Department of Agriculture, Gainesville, Florida, United States; 2 Southwest Florida Research and Education Center, University of Florida, Immokalee, Florida, United States; 3 Agriculture Research Service, United States Department of Agriculture, Ft. Pierce, Florida, United States

**Keywords:** ACP, Asian citrus psyllid, Morphometrics, Host plant, Huanglongbing

## Abstract

The Asian citrus psyllid (ACP), *Diaphorina citri* Kuwayama, is one of the most serious citrus pests worldwide due to its role as vector of huanglongbing or citrus greening disease. While some optimal plant species for ACP oviposition and development have been identified, little is known of the influence of host plants on ACP size and shape. Our goal was to determine how size and shape of ACP wing and body size varies when development occurs on different host plants in a controlled rearing environment. ACP were reared on six different rutaceous species; *Bergera koenigii*, *Citrus aurantifolia*, *Citrus macrophylla*, *Citrus maxima*, *Citrus taiwanica* and *Murraya paniculata*. Adults were examined for morphometric variation using traditional and geometric analysis based on 12 traits or landmarks. ACP reared on *C. taiwanica* were consistently smaller than those reared on the other plant species. Wing aspect ratio also differed between *C. maxima* and *C. taiwanica*. Significant differences in shape were detected with those reared on *M. paniculata* having narrower wings than those reared on *C. macrophylla*. This study provides evidence of wing size and shape differences of ACP based on host plant species which potentially may impact dispersal. Further study is needed to determine if behavioral and physiological differences are associated with the observed phenotypic differences.

## Introduction

The Asian citrus psyllid (ACP), *Diaphorina citri* Kuwayama, transmits the phloem-limiting bacterium, *Candidatus* Liberibacter asiaticus (*C*Las) (Grafton-Cardwell, Stelinski & Stansly, 2013) which causes a serious disease of citrus known as Huanglongbing (Bové, 2006). Currently, ACP exists in every major citrus producing region of the world except Europe, South Africa (which is dominated by another psyllid *Trioza erytreae* (Del Guercio)), and Australia (Narouei-Khandan et al., 2016). However, these areas do contain large citrus growing regions with climates suitable for establishment of ACP and *C*Las (Narouei-Khandan et al., 2016). First discovered in the United States (Florida) in 1998 (Halbert, 1998), the ACP is thought to have originated from southwestern Asia based on both morphometric (Lashkari, Hentz & Boykin, 2015) and mitochondrial cytochrome oxidase haplotype correspondence (Boykin et al., 2012). The success of ACP spreading from the place of origin (India) (Hall, 2008) indicates an ability to adapt to different environmental conditions existing throughout citrus growing regions of the world.

ACP can reproduce on most citrus and citrus relatives in the plant Family Rutaceae. In a study of 84 different species of cultivated citrus only *Casimiroa edulis* in the Subfamily Toddalioideae was completely uninfested with eggs, nymphs or adult ACP (Westbrook et al., 2011). Several other species tested, such as the rootstock *Poncirus trifoliate* possessed low numbers of ACP eggs, nymphs, and adults (Westbrook et al., 2011). Provision of high quality laboratory-reared ACP is imperative for many research projects and species used for rearing include *Bergera koenigii* (L.) (curry tree) (Simmons et al., 2013; Martini, Hoyte & Stelinski, 2014), *Citrus medica* L. (Hall & Hentz, 2016), *Citrus macrophylla* Wester (alemow) (Hall & Richardson, 2013) and *Murraya paniculata* (orange jasmine) (Hall, Lapointe & Wenninger, 2007; Paris et al., 2015). Recently Hall & Hentz (2016) reported on differences in development times and times to peak emergence in ACP reared on five different species of Rutaceae under greenhouse conditions. Adult ACP collected from different host plants in the field in Mexico were reported to exhibit morphometric variation (García-Pérez et al., 2013), although the role of biotic and abiotic factors in this variation was unclear.

Morphometric analysis provides a tool to evaluate phenotypic variation that results from a range of biotic and abiotic factors (Daly, 1985). Traditional morphometric analysis of size and size ratios has been the classical approach for quantifying variation in biological specimens. It is used to determine instar, and to compare genetic, environmental and phenotypic variation (Daly, 1985). A newer approach, geometric morphometric analysis, provides a mechanism to evaluate shape independent of size through the use of landmarks on two- and three-dimensional surfaces (Rohlf & Slice, 1990; Bookstein, 1991; Bookstein, 1996; Rohlf & Marcus, 1993; Dryden & Mardia, 1998). Geometric morphometric analysis has provided insight into patterns of morphological variation associated with *Musca domestica* L. wild populations and laboratory colonies (Ludoški et al., 2014), wild sandfly populations (Santos et al., 2015), the discrimination of four species of *Culex* mosquitoes (Laurito, Almirón & Ludueña-Almeida, 2015) and synonymy of two *Bactrocera* species (Schutze et al., 2015). Geometric morphometric analysis has also been instrumental in visualizing and comparing morphometric variation in relation to factors such as competition of sympatric species (Adams & Rohlf, 2000), environmental variables such as temperature and rainfall (Benítez et al., 2014), temporal variation (Drake & Klingenberg, 2008), and geographic variation (Lashkari et al., 2013). Host plant species have been reported to affect morphometric measurements of insects such as the potato psyllid, *Bactericera cockerelli* (Šulc) (Vargas-Madríz et al., 2013), *Bemisia tabaci* (Gennadius) (Bethke, Paine & Nuessly, 1991; Thomas et al., 2014), *Helicoverpa armigera* (Hűbner) (Khiaban et al., 2010), the butterfly *Heliconius erato* (Jorge et al., 2011), the winged/wingless morphs of male pea aphids, *Acyrthosiphon pisum* (Harris) (Frantz, Plantegenest & Simon, 2010), and ACP collected from Mexico (García-Pérez et al., 2013).

The objective of this study was to assess the effect on morphometric variation of ACP reared on different rutaceous host plant species under controlled environmental conditions. The choice of *Citrus* varieties suboptimal for ACP in key areas of the groves has been proposed as a possible component of an ACP management strategy (Alves, Diniz & Parra, 2014). Use of both approaches to morphometric analysis facilitated relating results to prior studies of ACP that used both traditional (Lashkari, Hentz & Boykin, 2015; García-Pérez et al., 2013) and geometric (Lashkari et al., 2013) morphometric analyses. Additionally, both approaches aided in fully applying literature data for interpreting results, particularly regarding dispersal.

## Materials and Methods

### Host plants and insect rearing

Plants were grown from seed starting on February 19, 2013. Both plants and ACP were reared in a conventional greenhouse supplied with an evaporative cooling system at the USDA-ARS Horticultural Research Laboratory in Ft. Pierce FL (Hall & Hentz, 2016). ACP colonies were maintained on six different host plant species: *Bergera koenigii* L. (curry tree), *Citrus aurantifolia* (Christm.) Swing (Mexican lime), *Citrus macrophylla* (alemow), *Citrus maxima* (Berm. F.) Merr. (pomelo), *Citrus taiwanica* Tan and Shim. (Nanshao Daidai sour orange), and *Murraya paniculata* (orange jasmine). All six species were known to support all life stages of ACP (Westbrook et al., 2011). Plants were grown from seed in steamed potting mix (Pro-Mix BX, Premier Horticulture, Inc., Quakertown, PA, USA) and fertilized weekly with water-soluble fertilizer mix (20N-10P-20K) (Peters Professional, The Scotts Company, Marysville, OH, USA). ACP were originally obtained from a USDA-ARS colony routinely maintained on *C. macrophylla*. Colonies were established by trimming plants to stimulate flush for oviposition and nymphal development. Two plants with flush were placed into each of several cages (BugDorm-2, BD2120F, MegaView Science Education Services Co., Ltd, Taichung, Taiwan), into which some adults were introduced and allowed to oviposit for 2 days and then removed. Resulting immature ACP were allowed to develop to the adult stage. The ACP colony was started on August 19, 2014. Adult ACP began emerging September 2, 2014. Adults for analysis were collected upon emergence from the colonies starting on September 5, 2014 through September 24, 2014. During the development time of the ACP the mean daily temperature in the greenhouse ranged from 25.8–28.2 °C, placed into labeled microcentrifuge tubes and held in a freezer until processed.

### Preparation and digitization of specimens

Only female ACP were utilized for morphometric analysis to avoid gender-related differences confounding the data analysis as male traits are generally shorter in length than females (García-Pérez et al., 2013). Color morphs of ACP were classified as either blue/green or gray/brown based on the abdominal color of newly emerged adults (Wenninger & Hall, 2008). Many ACP possessed yellow ova or reproductive structures on the blue/green or gray/brown abdomens, yet this color was not considered in the analysis as it occurs on both male and females and is thought to be related to the age of the ACP (Wenninger & Hall, 2008). All ACP samples were measured after mounting the body, right forewing and tibia onto glass slides using 10% bovine serum albumin solution. Slide-mounted specimens were digitally photographed using ultra-small high-performance zoom lens (Model: VH-Z100R; Keyence, Osaka Japan) at 100X magnification and a free angle observation system (VH-S30K; Keyence). A 1 mm scale was used to calibrate body length measurements. Measurements of the digital images were made on a computer using ImageJ software (Version 1.47) (Schneider, Rasband & Eliceiri, 2012).

### Traditional morphometrics

**Figure 1:**
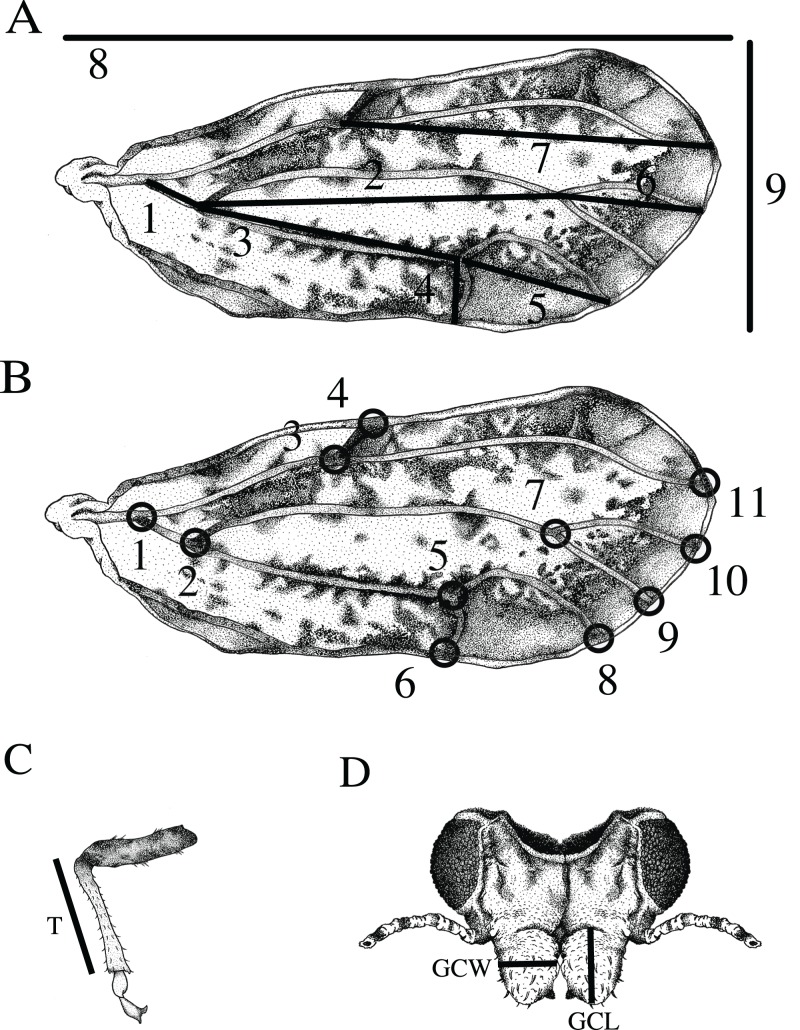
Features measured for morphometric analysis. (A) Wing measurements used for traditional morphometrics included: 1) length of the M+Cu_1_ vein, 2) the M vein, 3) the Cu_1_ vein, 4) the Cu_1b_ vein, 5) the Cu_1a_ vein, 6) the M_1+2_ vein, 7) the R_s_ vein, 8) overall wing length and 9) wing width. (B) Eleven landmarks on the wing used for geometric morphometric analysis as indicated by black circles. Body measurements used for traditional morphometrics. (C) Length of the tibia (T). (D) Length of the left genal comb (GCL) and width of the right genal comb (GCW). (Drawing by Xavier Moss).

Wing and vein nomenclature are based on Hodkinson & White (1979). Measurements of twelve standard morphological traits were obtained from the forewing, tibia, and genal process (Fig. 1): (1) wing length measured as the distance between the proximal end of the C+Sc vein to the wing apex, (2) wing width measured as the distance between the apex of the anterior to the apex of the posterior forewing, (3) tibia length (T) measured from the apex of tibia where it connects to the femur to the most distal part of the femur where it connects to the tarsus, (4) length of genal process (GCL) measured from the base to the tip of the apex, (5) width of genal process (GCW) measured at the widest part of the genal process, (6) length of the M+Cu_1_ vein, (7) length of the Cu_1_ vein (8) length of the Cu_1b_ vein, (9) length of the Cu_1a_ vein, (10) length of the M vein, (11) length of the M_1+2_ vein, and (12) length of the R_s_ vein. Vein measurements (6–12) were obtained by calculating the interlandmark distance used in geometric morphometrics (see below). The distances were calculated using the Pythagorean theorem.

(1) $${a^2} + {b^2} = {c^2}$$

Wing aspect ratio was calculated using the ratio of wing length to wing width. Wing loading was calculated by the ratio of tibia length to wing length. Tibia length is considered a correlate of the overall size of the organism (Kjaersgaard et al., 2015). Each measurement was repeated twice, and the average was used to reduce measurement error.

### Geometric morphometrics

Wing shape was quantified based on a set of 11 homologous landmarks (*x* and *y* coordinates in a Cartesian space) consisting of intersections between wing veins or wing veins and the wing margin recorded from the right forewing of each specimen (Fig. 1) (Bookstein, 1991; Rohlf & Marcus, 1993). Landmarks were digitized using ImageJ software with the software plugin PointPicker (http://bigwww.epfl.ch/thevenaz/pointpicker/) (Thévenaz, 2013). Measurement error as a result of landmark placement was quantified for each specimen by making two sets of landmarks and using the average for comparisons of ACP reared on different host plants.

### Data analysis

Linear measurements using traditional morphometrics were analyzed with the null hypothesis that there were no significant differences among measurements of ACP reared on different plants. All linear measurements were log-transformed for analysis. To compare each trait between host plants, data were analyzed using one-way analysis of variance (ANOVA) followed by mean separation with Tukey HSD test (*P* ≤ 0.05) contingent on a significant effect using R Studio (Racome, 2011) and R Statistical software (R Development Core Team, 2009). The following R packages were used for the data analysis: Plotrix (Lemon, 2006), Agricolae (de Mendiburu, 2015), Reshape (Wickham, 2007), Lattice (Sarkar, 2008), and Vegan (Oksanen et al., 2015). Ordination of the data using principal components analysis was used to determine which traits contributed the most variability. A second ordination technique, canonical variate analysis, was used to examine group differences and included a cross-validation confusion matrix. Group means were compared via multivariate analyses using Paleontological Statistics (PAST) v. 3.04 (Hammer, Harper & Ryan, 2001). A posteriori cross-validation and jackknife procedures were applied to determine the ability of canonical variate analysis to assign individuals to the correct group (Viscosi & Cardini, 2011). Dendrograms depicting squared Euclidean distances of size data between groups were plotted using unweighted pair group method with arithmetic mean (UPGMA) hierarchical cluster analysis (Sneath & Sokal, 1973) using PAST software (Hammer, Harper & Ryan, 2001). There were a total of 122 specimens used for the size and shape analysis (*M. paniculata = 20, C. taiwanica = 20, C. macrophylla = 19, C. maxima = 23, C. aurantifolia = 21, B. koenigii = 19*), however, some tibia and genal combs were lost and less samples were available to conduct the multivariate analysis of the linear measurements (*M. paniculata = 13, C. taiwanica = 15, C. macrophylla = 9, C. maxima = 16, C. aurantifolia = 16, B. koenigii = 16*).

Shape data (as Procrustes coordinates) were obtained using Procrustes superimposition, which removed size, position and orientation data and only extracted variation from shape (Dryden & Mardia, 1998; Rohlf & Slice, 1990). Geometric shape variation was analyzed based on Procrustes coordinates using multivariate techniques in MorphoJ v.1.04a software (Klingenberg, 2011). Shape variation from allometry (Gould, 1966; Klingenberg, 1996) was removed by using the residuals of the regressed log-transformed standard size measurement (the centroid) in shape analysis. The centroid is an estimator of size based on the square root of the sum of the distances of each landmark from the center of the landmark grouping (Parsons, Robinson & Hrbek, 2003). Ordination technique principal component analysis was undertaken to analyze patterns in the data. Discriminant function analysis was used for groups of two and canonical variate analysis for larger groups (Campbell & Atchley, 1981) with significant differences determined by permutations (10,000 rounds) to determine Mahalanobis distances between means. Cross validation of the discriminant functional analysis or canonical variate analysis was done using a confusion matrix that determines the accuracy of classifying the individuals to the proper group based on the discriminant function analysis (Viscosi & Cardini, 2011). Dendrograms depicting Procrustes distance of shape data between groups were plotted using UPGMA hierarchical cluster analysis (Sneath & Sokal, 1973) using PAST software (Hammer, Harper & Ryan, 2001). Differences between the average shape of ACP reared on all plants and the shape of ACP reared on a particular species were constructed using the MorphoJ software and provided a visual comparison of effects of each treatment on landmark values (Klingenberg, 2011).

## Results

### Traditional morphometrics

**Figure 2:**
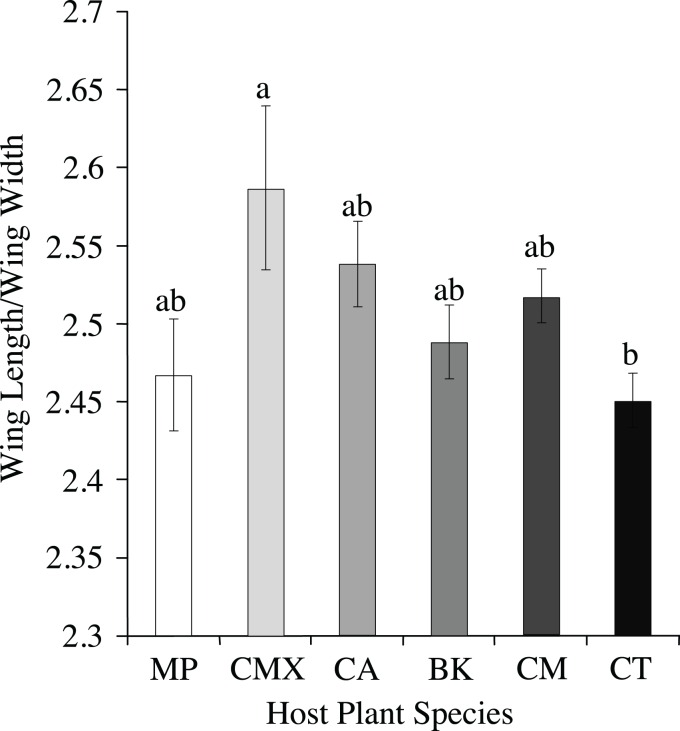
Wing aspect ratios (wing length/wing length) (mean ± SE) for ACP reared on different host plant species (BK, *Bergera koenigii*; CA, *Citrus aurantifolia*; CM, *Citrus macrophylla*; CMX, *Citrus maxima*; CT, *Citrus taiwanica*; MP, *Murraya paniculata*). Columns with different letters are significantly different.

Most ACP obtained from *C. taiwanica* were smaller than ACP obtained from other plant species. All traits measured differed significantly among host plant species except for wing width, and the lengths of the M+Cu_1_ and Cu_1b_ veins (Table 1). Adult psyllids reared on *C. aurantifolia* were consistently larger compared to psyllids raised on *C. taiwanica* but similar to those reared on all other hosts tested. ACP reared on *C. taiwanica* were similar to those reared on *B. koenigii* except for a smaller genal comb length. The greatest range of size difference was observed with the genal comb length which was largest for ACP reared on *M. paniculata*, intermediate from *B. koenigii* and smallest from *C. taiwanica*. When all ACP reared from host plants were combined and only color morph considered, the only traits that differed were M+Cu_1_ (t = −2.34, *P* = 0.02) and M (t = −1.99, *P* = 0.05) veins of the gray/brown colored ACP which were significantly smaller than the blue/green colored ACP (data not presented). There were also significant differences among ACP reared on different plants in terms of wing aspect ratio (wing length/wing width) (F = 2.51; df = 5.89; *P* < 0.04), but not wing loading (tibia length/wing length) (F = 0.80; df = 5.89; *P* = 0.55). ACP reared on *C. taiwanica* had significantly lower wing aspect ratio compared to ACP reared on *C. maxima* (Fig. 2). All other host plants produced ACP in the intermediary range.

**Table 1:** Size of morphological traits (mm ± SE) measured from Asian citrus psyllid females reared from different host plants. *P*-values are provided for ANOVA analysis comparisons of each trait between plant species.

Traits (length)	*B. koenigii*	*C. aurantifolia*	*C. macrophylla*	*C. maxima*	*C. taiwanica*	*M. paniculata*	*P-value*
Wing length	1.95 ± 0.01ab	2.04 ± 0.01a	2.00 ± 0.02a	2.04 ± 0.01a	1.86 ± 0.01b	2.04 ± 0.01a	< 0.0001
Wing width	0.79 ± 0.01	0.79 ± 0.00	0.80 ± 0.01	0.79 ± 0.01	0.76 ± 0.01	0.83 ± 0.01	0.06
Tibia	0.41 ± 0.01ab	0.44 ± 0.01a	0.42 ± 0.01ab	0.43 ± 0.01a	0.39 ± 0.01b	0.42 ± 0.01ab	0.01
Genal comb length	0.14 ± 0.02b	0.16 ± 0.01ab	0.16 ± 0.01ab	0.15 ± 0.01ab	0.11 ± 0.02c	0.17 ± 0.01a	< 0.0001
Genal comb width	0.11 ± 0.01ab	0.11 ± 0.01a	0.10 ± 0.00ab	0.11 ± 0.01a	0.10 ± 0.01b	0.11 ± 0.01a	< 0.0001
M+Cu_1_	0.25 ± 0.01b	0.26 ± 0.01b	0.26 ± 0.00b	0.25 ± 0.02ab	0.23 ± 0.01a	0.26 ± 0.01b	0.05
Cu_1_	0.62 ± 0.01ab	0.65 ± 0.01a	0.64 ± 0.01a	0.66 ± 0.01a	0.58 ± 0.00b	0.66 ± 0.01a	< 0.0001
M	0.83 ± 0.01ab	0.89 ± 0.01a	0.87 ± 0.01a	0.89 ± 0.01a	0.79 ± 0.01b	0.87 ± 0.01a	< 0.0001
Cu_1b_	0.17 ± 0.01	0.17 ± 0.01	0.16 ± 0.00	0.15 ± 0.02	0.15 ± 0.01	0.16 ± 0.01	0.17
Cu_1a_	0.44 ± 0.01ab	0.45 ± 0.00a	0.44 ± 0.00ab	0.44 ± 0.01ab	0.42 ± 0.00b	0.45 ± 0.01ab	0.03
M_1+2_	0.54 ± 0.01ab	0.55 ± 0.01a	0.54 ± 0.01a	0.54 ± 0.01a	0.49 ± 0.01b	0.56 ± 0.01a	0.005
R_s_	1.12 ± 0.01ab	1.17 ± 0.01a	1.15 ± 0.01a	1.15 ± 0.01a	1.05 ± 0.00b	1.17 ± 0.01a	0.0002

**Note:**

Different letters within a row designate significantly different means (α < 0.05) (Tukey HSD) between plant species.

The first two principal components accounted for 93.6% of the total variation observed among species (PC1 = 90.1%, PC2 = 3.5%). The greatest contributions to the first principal component were the M_1+2_ (0.49) and R_s_ (0.49) veins with moderate correlation (0.39) from the length of the M vein (Table 2). In the second principal component, the Cu_1b_ vein was strongly correlated (0.78), while the length of the genal comb length was moderately correlated (0.46) (Table 2). Analysis by permutational multivariate analysis of variance (PERMANOVA) revealed significant differences between ACP reared on different host plant species (N_perm_ = 10,000; *P* < 0.001; F_5,80_ = 5.15).

**Table 2:** Coefficients of the first two principal components (PC1 and PC2) of the principal component analysis of traditional morphometrics of ACP reared on different host plant species.

Trait	PC1	PC2
Wing length	0.0278	0.1181
Wing width	0.0223	0.1408
Tibia	0.0199	0.0947
Genal comb length	0.0826	0.4593
Genal comb width	0.0328	0.2156
M+Cu_1_	0.3780	−0.1465
Cu_1_	0.3543	0.0702
M	0.3857	0.0573
Cu_1b_	0.0784	0.7789
Cu_1a_	0.3033	0.1219
M_1 + 2_	0.4854	−0.1992
R_s_	0.4893	−0.0945
Eigenvalues	0.224	0.009
Proportions	90.052	3.53

**Figure 3:**
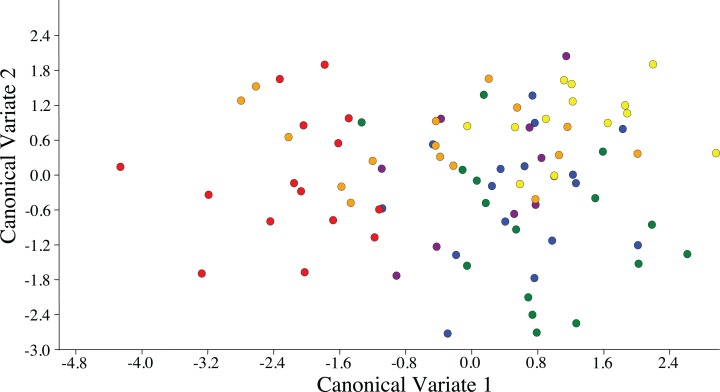
Scatterplot depicting the first two canonical variates of a canonical variate analysis of distance variables of female ACP reared on different host plant species (*Bergera koenigii* = orange dot, *Citrus aurantifolia* = blue dot, *Citrus macrophylla* = green dot, *Citrus maxima* = purple dot, *Citrus taiwanica* = red dot, *Murraya paniculata* = yellow dot).

Visualization of the canonical variate analysis scatterplot did not demonstrate complete separation among plant species. However, ACP reared on *C. taiwanica* were completely separated from ACP reared on *M. paniculata* and minimal overlap with those reared on *C. aurantifolia*, *C. macrophylla* and *C. maxima* (Fig. 3). The first two canonical variables (CV1 and CV2) explained 86.4% of the total variance. Lengths of the Cu_1_ vein, M vein, M_1+2_ vein, and R_s_ vein wing were the highest contributors for the first canonical axes (CV1) (Table 3).

**Table 3:** Coefficients for the first two canonical variates (CV1 and CV2) for analysis of traditional morphometrics of ACP reared on different host plant species.

Trait	CV1	CV2
Wing length	0.0111	−0.0019
Wing width	0.0074	0.0105
Tibia	0.0110	−0.0136
Genal comb length	0.0448	0.0165
Genal comb width	0.0150	−0.0048
M+Cu_1_	0.0663	−0.0207
Cu_1_	0.0669	−0.0170
M	0.0702	−0.0266
Cu_1b_	0.0078	0.0041
Cu_1a_	0.0512	−0.0179
M_1+2_	0.0850	−0.0215
R_s_	0.0868	−0.0249
Eigenvalues	1.7646	0.2808
Proportions	74.51	11.86

**Figure 4:**
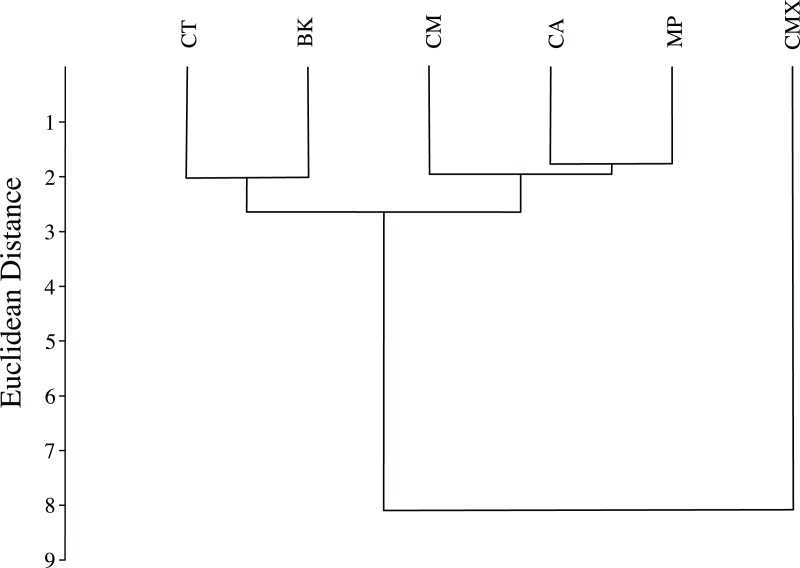
Dendrogram formed by means of the UPGMA method using squared Euclidean distances of ACP reared on different host plant species (BK, *Bergera koenigii*; CA, *Citrus aurantifolia*; CM, *Citrus macrophylla*; CMX, *Citrus maxima*; CT, *Citrus taiwanica*; MP, *Murraya paniculata*).

ACP reared on *C. taiwanica* exhibited larger Malahanobis distances compared to ACP reared on the other plants (Table 4). A posteriori classification summary assigned 61% of ACP into the correct host plant based on traditional morphometric measurements. A subsequent, jacknifed cross-validation resulted in correct assignment of 33% of ACP to the proper host plant using traditional morphometrics (Table 5). ACP reared on *C. taiwanica* had the lowest misclassification, 23 and 27% for posteriori and jacknife cross validation, respectively. The a posteriori classification error rate was reduced to 8% by randomly averaging the samples into groups of three, while the jacknifed cross-validation was not improved by averaging. A high error rate is expected in a sample of the same species where subtle intraspecific differences are examined. The host plant association is better able to differentiate ACP with respect to host plant species based on the average measurements of the group than to assign individuals to the correct host plant species on which they were reared. Cluster analysis using squared Euclidean showed ACP reared on *C. maxima* separate from the others in a single branch, while ACP reared on the remaining plants clustered into a second multilevel branch (Fig. 4).

**Table 4:** Summary of Mahalanobis distances based on traditional morphometrics among female ACP reared on different host plants.

Species	BK	CA	CM	CMX	CT	ME
BK						
CA	2.2553					
CM	1.3333	1.0648				
CMX	4.5362	1.2895	2.6166			
CT	4.4044	8.5432	6.7734	9.6029		
MP	4.8336	3.5896	4.1344	3.7947	13.281	

**Notes:**

BK, *Bergera koenigii*; CA, *Citrus aurantifolia*; CM, *Citrus macrophylla*; CMX, *Citrus maxima*; CT, *Citrus taiwanica*; MP, *Murraya paniculata.*

**Table 5:** A posteriori classificatory summary for discriminant analysis of traditional morphometric measurements for ACP reared on different host plants.

Cultivar	Total number of observations	Classified correctly		Misclassified		Number of specimens misclassified					
		Number	%	Number	%	BK	CA	CM	CMX	CT	MP
BK	16	10	63	6	20	–	0	0	1	3	2
CA	16	7	44	9	24	3	–	1	4	0	1
CM	9	4	44	5	37	2	2	–	0	0	1
CMX	16	8	50	8	24	1	4	0	–	1	2
CT	15	12	80	3	23	3	0	0	0	–	0
MP	13	11	85	6	10	0	1	1	0	0	–
Total	85	52	61	33	39	6	5	7	9	4	2
**Jackknife cross-validation**											
BK	16	2	12	14	88	–	2	2	1	5	4
CA	16	2	12	14	88	3	–	2	6	0	3
CM	9	1	11	8	89	1	2	–	1	1	3
CMX	16	3	19	13	81	1	5	2	–	2	3
CT	15	11	73	4	27	3	1	0	0	–	0
MP	13	8	62	5	38	1	1	2	1	0	–
Total	85	27	32	58	68	13	9	11	9	8	8

**Notes:**

BK, *Bergera koenigii*; CA, *Citrus aurantifolia*; CMX, *Citrus maxima*; CM, *Citrus macrophylla*; CT, *Citrus taiwanica*; MP, *Murraya paniculata*.

### Geometric morphometrics

Measurement error due to landmark placement was negligible as indicated by significantly less error for both the centroid and shape variation compared to the individual female ACP with respect to landmark placement (Table 6). As a result, not all ACP duplicating landmark placement was deemed necessary and more samples were collected instead of double measuring.

**Table 6:** Procustes ANOVA for the determination of error of the centroid and shape of female ACP with respect to landmark placement.

Effect	SS	MS	df	F	P-value
**Centroid**					
Individual	3.48551	0.03485	100	19.08	< 0.0001
Error 1	0.18474	0.00183	101		
**Shape**					
Individual	0.28587	0.00016	1,800	5.78	< 0.0001
Error 1	0.04998	0.00003	1,818		

**Figure 5:**
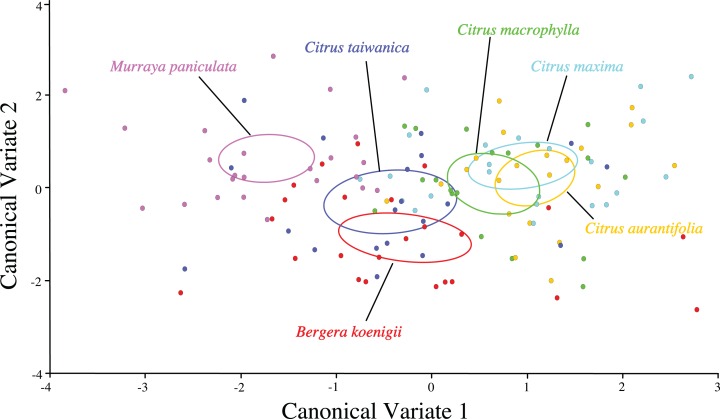
Scatterplot depicting the first two canonical variates of a canonical variate analysis of geometric morphometric data for wing shape variation of female ACP reared on different host plant species (*Bergera koenigii* = orange, *Citrus aurantifolia* = blue, *Citrus maxima* = green, *Citrus macrophylla* = purple, *Citrus taiwanica* = red, *Murraya paniculata* = yellow). Confidence ellipses (95%) represent means of wing shape.

Separation of confidence ellipses from ordination of the individuals in morphospace defined by the first two canonical variates demonstrated significant differences among ACP reared on different host plants based on the mean of ACP (Fig. 5). While ACP in the two covariates did not separate into discrete clusters, mean ellipses of wing shape formed three distinct clusters. Wing shape means and confidence ellipses did overlap for *C. taiwanica* and *B. koenigii* indicating similarity. There were no differences among wing shapes for ACP reared on *C. aurantifolia, C. maxima* and *C. macrophylla*. ACP reared on *M. paniculata* differed in wing shape from ACP reared on all of the other host plants. Mahalanobis distances indicated that differences in shape from the geometric morphometric analysis were evident (Table 7). Based on confidence ellipses (Fig. 5), ACP reared from *M. paniculata* differed in shape from ACP reared on all other host plants. ACP reared from *C. taiwanensis* differed from those reared on *C. macrophylla, C. maxima* and *C. aurantifolia* but not *B. koenigii*.

**Table 7:** Mahalanobis distances between populations of female ACP reared on different host plant species using geometric morphometric measurements. *P*-values for Hotellings *T*^2^ tests with 10,000 permutations are in parentheses.

	BK	CA	CM	CMX	CT	ME
BK						
CA	2.02 (0.001)					
CM	2.00 (0.002)	1.54 (0.08)				
CMX	2.06 (0.0001)	1.12 (0.76)	1.67 (0.01)			
CT	1.59 (0.17)	1.97 (0.001)	1.82 (0.002)	2.05 (0.0001)		
MP	2.26 (< 0.0001)	2.91 (< 0.0001)	2.69 (< 0.0001)	2.77 (< 0.0001)	1.97 (0.0001)	

**Notes:**

BK, *Bergera koenigii*; CA, *Citrus aurantifolia;* CM, *Citrus macrophylla*; CMX, *Citrus maxima*; CT, *Citrus taiwanica*; MP, *Murraya paniculata*.

**Figure 6:**
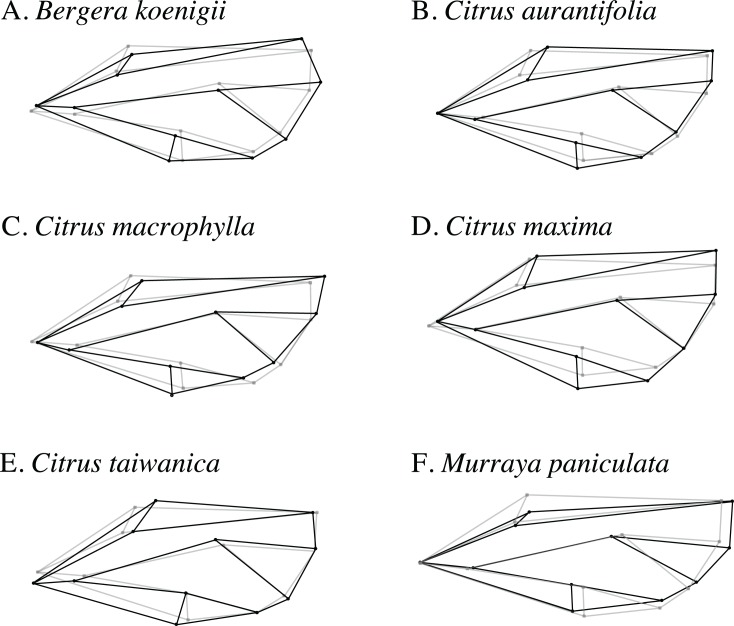
Wireframe visualizations of the average wing shape variation of the first principal component of female ACP reared on different host plants. The black lines of the wings occurring on each host plant show the shape changes from the average shape (gray line) of all ACP measured. (A) *Bergera koenigii,* (B) *Citrus aurantifolia,* (C) *Citrus macrophylla,* (D) *Citrus maxima,* (E) *Citrus taiwanica,* (F) *Murraya paniculata.*

Wireframe visualizations of the shape change of ACP wings reared on different host plant species provided visualization of differences in shape from each host compared to the overall average wing shape (Fig. 6). Wings from ACP reared on *M. paniculata* were clearly more narrow than average, whereas those from *C. taiwanica* and *B. koenigii* tended to be broader but were distinctly different from ACP reared on *C. maxima*, *C. macrophylla*, and *C. aurantifolia*.

**Figure 7:**
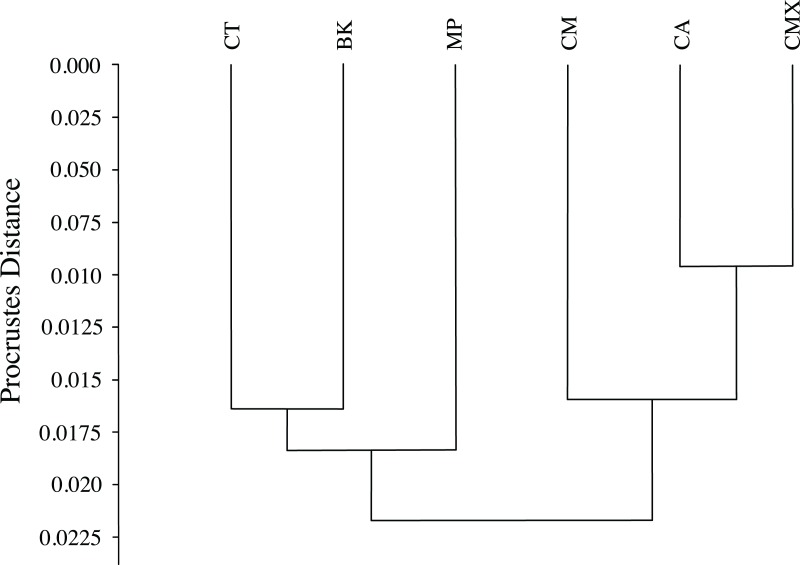
Dendrogram formed by means of the UPGMA method using Procrustes distances of female ACP reared on different host plant species. (BK, *Bergera koenigii*; CA, *Citrus aurantifolia*; CM, *Citrus macrophylla*; CMX, *Citrus maxima*; CT, *Citrus taiwanica*; MP, *Murraya paniculata*).

The graphical visualization of the Procrustes distances in a dendrogram indicated that ACP reared from *C. macrophylla*, *C. maxima* and *C. aurantifolia* separated from ACP reared on *B. koenigii*, *C. taiwanica*, and *M. paniculata* (Fig. 7). The most distinct ACP were those reared from *M. paniculata* and *C. macrophylla,* which formed a single branch on both trees (Figs. 4 and 7). When color morph of ACP was considered, the shape of gray/brown ACP were not significantly different from blue/green ACP according to Hotelling’s T^2^ test (*P* = 0.26; 10,000 permutations).

## Discussion

Rearing ACP on different host plant species clearly affected phenotypic variation in morphometric traits as determined by both traditional and geometric methods. Traditional morphometric measurements such as mean wing length and wing width of female ACP from our study were similar to those measured by Mathur (1975); EPPO (2005) and Chhetry, Gupta & Tara (2012). Other morphometric studies such as García-Pérez et al. (2013) and Pérez-Valencia & Moya-Raygoza (2015) recorded mean wing lengths of female ACP that were up to 2 mm larger than this study. The influence of different environmental variables such temperature or rainfall may play a role in the variation for ACP, for example along an elevational gradient (Pérez-Valencia & Moya-Raygoza, 2015). Phenotypic variation may also originate from genetic differences as eight haplotypes of ACP have been identified by Boykin et al. (2012). Another factor affecting our study may be a small sample size, which could have resulted in underestimation of effects.

Although blue/green ACP were reported to be larger and to fly farther than gray/brown ACP, no association was found between distance flown and wing length (Martini, Hoyte & Stelinski, 2014). However, abdominal color morphs of ACP were not associated with traditional morphometric variation in this study, in contrast to host plant type. The ability of individual ACP to alter abdominal color from gray/brown to blue/green (Wenninger & Hall, 2008) may have contributed to the general lack of significant difference detected in size or shape of morphometric traits.

ACP reared on *C. taiwanica* were smaller with broader wings and lower wing aspect ratio than those reared on the other host plants. However, there were no significant differences in wing loading among ACP reared on different host plants. ACP is a capable disperser between abandoned and managed citrus groves (Boina et al., 2009; Tiwari et al., 2010) and into habitats devoid of citrus (Hall & Hentz, 2011; Martini et al., 2013). Little is known of how morphometric variation affects dispersal capability of ACP and results from studies on other insects are inconsistent. Narrow wings or increased wing aspect ratios increased flight performance of the speckled wood butterfly, *Pararge aegeia* (Berwaerts, Van Dyck & Aerts, 2002). However, Dudley & Srygley (1994) reported that flight speed was negatively correlated with wing aspect ratio but positively correlated with wing loading in a study of 62 species of neotropical butterflies. In contrast, flight capability of house flies, *Musca domestica*, collected in three different European countries was not correlated with wing loading and only marginally correlated with wing aspect ratio (Kjaersgaard et al., 2015).

Tibial length is considered an effective correlate for overall body size (Kjaersgaard et al., 2015) and fecundity (Opp & Luck, 1986; Reeve, Fowler & Partridge, 2000). Tibia of ACP reared on *C. taiwanica* were shorter compared to other species. Lower production of ACP on new flush shoots of *C. taiwanica* and *M. paniculata* compared to *C. aurantifolia* was observed during a winter experiment although not in spring and summer (Hall & Hentz, 2016). Larger bodied ACP may be able to fly longer distances than their smaller bodied counterparts as is the case for *Scathophaga stercoraria* and *Aedes aegypti* (Kaufmann, Reim & Blanckenhorn, 2013).

Several vein measurements varied significantly among ACP reared on different host plants. Wing veins, particularly the R_s_ vein, were the most important loading factor in principal component analysis. Furthermore, landmark 7 (descriptive of vein pattern) was displaced to a larger degree from average on wings of ACP reared on *C. macrophylla* and *C. maxima*, which compared with the variation observed in the sizes of various wing veins in this study. Additionally, the R_s_ vein was an important loading factor along with wing, antennal and circumanal length in a principal component analysis in a study of ACP from different sites in Florida, Pakistan and Iran (Lashkari, Hentz & Boykin, 2015). In addition to veins, genal comb measurements were also an important source of variation in our study and the most important contributing factor for separation of the populations of field collected ACP obtained from specific host plants in Mexico (García-Pérez et al., 2013).

The most dramatic differentiation of wing shape was between *M. paniculata* and the other species tested. Conversely, wings of ACP reared on *M. paniculata* were narrow compared to the broad wings of ACP reared on other host plants. In a study of migrating dragonflies, broad wings were found to be associated with migration (Johansson, Söderquist & Bokma, 2009). Likewise, broad wings of two species of *Adialytus* braconids that parasitize arboricolous aphids were associated with strong flight ability in contrast to a narrow wing species that parasitized aphids in grasses and therefore was less dependent on flight (Stanković et al., 2015). Similarly, narrow wings of ACP reared on *M. paniculata* could be indicative of a decreased need for dispersal thanks to ideal quantities and qualities of flush tissue critical for oviposition and nymphal development. On the other hand, the fall form of the pear psyllid, *Cacopsylla pyricola*, has longer and narrower wings but is more dispersive then the summer form (Hodgson & Mustafa, 1984; Horton et al., 1994). The relationship between speed of movement and wing length measurements is not always direct (Dudley & Srygley, 1994). Additional studies are required to relate morphological differences in ACP to behavioral or physiological attributes that may affect dispersal or ultimately spread of pathogens through the landscape.

Generation time, oviposition levels, and survival rates are important factors related to host plant quality that effect population establishment (Alves, Diniz & Parra, 2014; Tsagkarakis & Rogers, 2010; Tsai & Liu, 2000; Nava et al., 2007). Several morphometric parameters including wing width, body length and antenna length of potato psyllids varied significantly when development occurred on different varieties of tomato plants (Vargas-Madríz et al., 2013). Morphometric variation in traits between ACP reared on different hosts may also result from differences in nutritional levels present among species. Nutrition of immature insects is known to significantly affect adult size (Nijhout, 2003). Both size and shape of wings have also been shown to vary with nutrition in the parasitic hemipteran, *Triatoma infestans* (Nattero et al., 2015). The mechanisms causing variation in wing size and shape in ACP could well reflect differences in nitrate or other nutrients available in different species of Rutaceae. For example, two varieties of sweet orange ‘Hamlin’ and ‘Valencia’ are known to vary in their sap nitrate content (Souza et al., 2012). While no morphometric comparisons based on nitrate content were made in this study, differences in chemotype and fertilization levels of the plant *Melaleuca quinquenervia* (Cav.) affected nymphal survivorship and development time of the psyllid, *Boreioglycaspis melaleuca* (Wheeler & Ordung, 2005), although weight was not affected.

There is a potential link between the host plant on which ACP nymph development occurs and ACP dispersal capacity as different shapes of ACP wings may be more or less ideal for long distance flight. The wing shape of ACP reared on *M. paniculata* was narrow compared to the other host plants examined. Narrower shaped wings are often associated with less dispersal (Stanković et al., 2015; Johansson, Söderquist & Bokma, 2009). Therefore, ACP reared on *M. paniculata* may produce forms less likely to disperse because they are developing on their ideal host plant. Furthermore, laboratory studies examining ACP learning in two choice assays indicated preference for host plants on which ACP were reared (Stockton et al., 2016). Dispersing ACP may disperse to and prefer citrus groves with the same cultivars on which they developed.

The observed phenotypic plasticity enhances our understanding of morphometric variability associated with host plants. Both the traditional and geometric analyses were effective for detection of differences in size and shape among ACP reared on different host plant species. Additional studies are needed to examine the behavioral capabilities of these different ACP phenotypes. Secondly, our study was largely confined to host plants of interest for research colonies and as such, these studies should be expanded to incorporate citrus species commonly used in commercial production.

## Supplemental Information

10.7717/peerj.2663/supp-1 Supplemental Information 1 Landmark values for traditional morphometric analysis.

10.7717/peerj.2663/supp-2 Supplemental Information 2 Landmark values for geometric analysis. Table S2. Landmark values for geometric morphometric analysis for Asian citrus psyllids reared on different host plants.

## Additional Information and Declarations

### Data Deposition

The following information was supplied regarding data availability: The raw data has been supplied as Supplemental Dataset Files.
